# Expression of Concern: Interferon and Ribavirin Combination Treatment Synergistically Inhibit HCV Internal Ribosome Entry Site Mediated Translation at the Level of Polyribosome Formation

**DOI:** 10.1371/journal.pone.0266498

**Published:** 2022-03-31

**Authors:** The PLOS ONE Editors

After this article [1] was published, concerns were raised about similarities in some of the microscopy images in Figures 1A and 1D.

Specifically:

- In Figure 1A in this article, the 0 RBV (μg/ml), 100 IFN-α (IU/ml) panel appears similar to the 40 RBV (μg/ml), 10 IFN-α (IU/ml) panel; the 10 RBV (μg/ml), 0 IFN-α (IU/ml) panel appears similar to the 20 RBV (μg/ml), 10 IFN-α (IU/ml) panel; and the 20 RBV (μg/ml), 0 IFN-α (IU/ml) panel appears similar to the 20 RBV (μg/ml), 1000 IFN-α (IU/ml) panel.
- In Figure 1D in this article, the 0 RBV (μg/ml), 100 IFN-α (IU/ml) panel appears similar to the 10 RBV (μg/ml), 10 IFN-α (IU/ml) panel; and the 0 RBV (μg/ml), 250 IFN-α (IU/ml) appears similar to the 20 RBV (μg/ml), 250 IFN-α (IU/ml) panel.

Follow-up on these issues is ongoing; in the meantime, the *PLOS ONE* Editors issue this Expression of Concern.

In addition, please note that reference 16 in this article [1] has been retracted [2].
